# Nano-oxide thin films deposited via atomic layer deposition on microchannel plates

**DOI:** 10.1186/s11671-015-0870-y

**Published:** 2015-04-02

**Authors:** Baojun Yan, Shulin Liu, Yuekun Heng

**Affiliations:** Institute of High Energy Physics and State Key Laboratory of Particle Detection and Electronics, Chinese Academy of Sciences, 19B Yuquan Lu, Beijing, 100049 People’s Republic of China

**Keywords:** Microchannel plate (MCP), Atomic layer deposition (ALD), Thin film, High aspect ratio, Electrical performance

## Abstract

Microchannel plate (MCP) as a key part is a kind of electron multiplied device applied in many scientific fields. Oxide thin films such as zinc oxide doped with aluminum oxide (ZnO:Al_2_O_3_) as conductive layer and pure aluminum oxide (Al_2_O_3_) as secondary electron emission (SEE) layer were prepared in the pores of MCP via atomic layer deposition (ALD) which is a method that can precisely control thin film thickness on a substrate with a high aspect ratio structure. In this paper, nano-oxide thin films ZnO:Al_2_O_3_ and Al_2_O_3_ were prepared onto varied kinds of substrates by ALD technique, and the morphology, element distribution, structure, and surface chemical states of samples were systematically investigated by scanning electron microscopy (SEM), energy-dispersive X-ray spectroscopy (EDS), X-ray diffraction (XRD), and X-ray photoemission spectroscopy (XPS), respectively. Finally, electrical properties of an MCP device as a function of nano-oxide thin film thickness were firstly studied, and the electrical measurement results showed that the average gain of MCP was greater than 2,000 at DC 800 V with nano-oxide thin film thickness approximately 122 nm. During electrical measurement, current jitter was observed, and possible reasons were preliminarily proposed to explain the observed experimental phenomenon.

## Background

Microchannel plate (MCP) is a thin glass plate with thickness of about 0.5 mm consisting of several millions of pores of a cylinder geometry with a 4 ~ 25 μm diameter and with a bias angle usually 5° ~ 13° to the normal of the plate surface; the open area ratio of the plate is up to 60%, and the high aspect ratio in each pore is about 20:1 to 100:1. MCP usually as a kind of electron multiplied device can be used in many scientific applications, such as microchannel plate photomultipliers (MCP-PMT), night vision devices, electron microscopy, and fluorescent electron imager [1-5]. Traditional MCP is made of lead silicate glass, and the production process is complex [6]. However, a new type of MCP is made of borosilicate glass in the form of hollow tubes and then fabricated in a typical drawing/stacking/fusing/slicing process, without extensive hydrogen reduction chemical processing [7].

**Figure 1:**
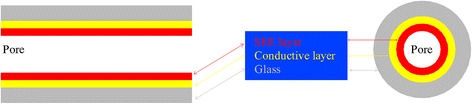
**Structure schematic diagram of a pore of ALD-MCP.**

Generally, tandem nano-oxide layers including zinc oxide doped with aluminum oxide (ZnO:Al_2_O_3_) as conductive layer and aluminum oxide (Al_2_O_3_) as secondary electron emission (SEE) layer shown in Figure 1 were deposited on the traditional MCP pores to conduct electron multiplication function. However, all regular nano-oxide thin film deposition methods, such as plasma-enhanced chemical vapor deposition (PECVD), molecular beam epitaxy (MBE), magnetic sputtering, electron beam evaporation, and pulsed laser deposition (PLD) [8-13], cannot grow uniform nano-oxide thin films in a high aspect ratio structure. So far, the only effective approach growing high-quality thin films is atomic layer deposition (ALD) technique based on sequential self-terminating gas-solid reactions invented both by the group of Professor Aleskovskii in the 1960s in the Soviet Union [14] and by Suntola and co-workers in the 1970s in Finland [15]. The typical process of Al_2_O_3_ deposited by ALD has been described by two successive ‘half reactions’ presented in Equations 1 and 2 [16]:

1 $$ \mathrm{A}:\ \mathrm{Substrate}\hbox{-} \left(\mathrm{O}\mathrm{H}\right)*+\mathrm{A}\mathrm{l}{\left({\mathrm{CH}}_3\right)}_3\to \mathrm{Substrate}\hbox{-} \mathrm{O}\hbox{-} \mathrm{A}\mathrm{l}{\left({\mathrm{CH}}_3\right)}_2*+{\mathrm{CH}}_4\uparrow $$

2 $$ \mathrm{B}:\ \mathrm{Substrate}\hbox{-} \mathrm{O}\hbox{-} \mathrm{A}\mathrm{l}{\left({\mathrm{CH}}_3\right)}_2*+2{\mathrm{H}}_2\mathrm{O}\to \mathrm{Substrate}\hbox{-} \mathrm{O}\hbox{-} \mathrm{A}\mathrm{l}{\left(\mathrm{O}\mathrm{H}\right)}_2*+2{\mathrm{CH}}_4\uparrow $$

**Figure 2:**
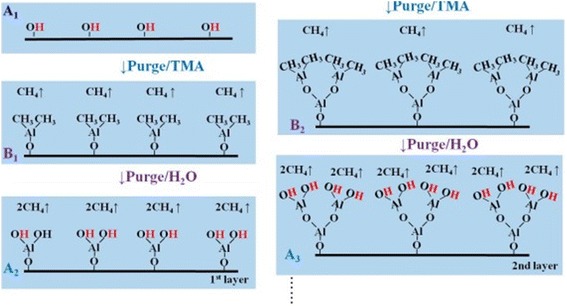
**Schematic illustration of ALD Al**_**2**_ **O**_**3**_ **growth process.** First layer sequence: on substrate surface **(A**_**1**_ **)**, pulsing TMA after purge **(B**_**1**_ **)**, then pulsing H_2_O after purge **(A**_**2**_ **)**. Second layer sequence: pulsing TMA after purge **(B**_**2**_ **)**, then pulsing H_2_O after purge **(A**_**3**_ **)**.

The equation showing substrate surface switches from methyl-terminated to hydroxyl-terminated and vice versa is well known by infrared measurements [17] and illustrated in Figure 2.

One can see from Figure 2 that the substrate surface is initially covered with hydroxyl (OH) groups which react with trimethylaluminum (TMA) to deposit a mono-layer of aluminum-methyl groups and give off methane (CH_4_) as a byproduct. This new surface is exposed to water regenerating the initial hydroxyl-terminated surface and again releasing CH_4_. Then, one mono-layer of Al_2_O_3_ is deposited on the surface. And Figure 2 also shows the steric hindrance of the ligands which is a factor to cause the saturation of the surface, and another factor is the number of reactive surface site (not shown in Figure 2) [18-20]. Due to the absorbing mechanism in ALD method, the saturation of the absorbed precursor is very important for the growth rate of the thin films. For fabricating composite films, such as ZnO:Al_2_O_3_ alloys, by adjusting the ALD pulse sequence, ‘etching’ phenomenon has been observed that many of the ZnO:Al_2_O_3_ samples prepared in a viscous flow ALD reactor showed lower than expected Zn film content and were thinner than predicted by the ALD growth rates of the pure ZnO and Al_2_O_3_ films [21-23]. The Zn deficiency may result from the etching of Zn by the TMA during the Al_2_O_3_ cycles by quartz crystal microbalance measurements, and the lower than expected film thickness is caused by a reduced initial growth rate for ZnO ALD on Al_2_O_3_ surface and vice versa. So, precisely controlling the thickness and composition of ZnO:Al_2_O_3_ films is difficult, especially on complex substrate if a viscous flow reactor is utilized.

The optimization of ALD-MCP with conductive layers and SEE layers is a complicated process because nano-oxide thin film performance depending upon a lot of ALD process parameters was influenced by the existence of an ‘etching’ phenomenon. The conductive and SEE layers as nano-engineered thin films were shown previously to be successfully and uniformly deposited by Sullivan et al. onto non-lead glass MCPs [24,25] and plastic MCPs [26]. And the Argonne National Laboratory has systematically studied conductive layers on a broad range of oxides, such as ZnO:Al_2_O_3_, W:Al_2_O_3_, and MoO_3 − *x*_:Al_2_O_3_ [27,28], and characterized the SEE properties for MgO, Al_2_O_3_, and multilayered MgO/TiO_2_ structures to serve as electron emissive layers in the channels of the MCPs [29]. However, the relationship of electrical performance of an MCP device as a function of nano-oxide thin film thickness has not been reported.

In this study, ZnO:Al_2_O_3_ as conductive layer and Al_2_O_3_ as SEE layer were prepared by ALD technique in the pores of the MCP with 33 mm in diameter. The morphology, element distribution, structure, and surface chemical states of ALD-deposited oxide thin films and the electrical performance of ALD-MCP were systematically investigated.

## Methods

A commercial hot-wall atomic layer deposition system was used to prepare nano-oxide thin films onto MCP, glass, and polished n-Si (100) substrates. The glass substrates were ultrasonically cleaned in an ethanol/acetone solution and then rinsed in deionized water. The polished Si substrates were dipped in hydrofluoric acid for 30 s and then placed in an ALD chamber waiting for deposition. The bare MCPs (thickness = 0.5 mm, pore size = 20 μm, aspect ratio = 25, bias angle = 8°) were heated to 350°C for 2 h prior to growing nano-oxide thin films. According to the complex structure of MCP, controlling the thickness and composition of ZnO:Al_2_O_3_ films on MCP is harder than controlling those on planar substrate [21,22]. During ALD-ZnO:Al_2_O_3_ alloy film fabrication process with certain Zn content, the ‘etching’ phenomenon is unavoidable, and the actual thickness and composition of films are lower than the expected values. By our *ex situ* measurements, the actual results can match the ‘expected’ one with good repeatability. And two approaches were adopted to make thickness and composition as far as possible be uniformly distributed in the pores of MCP. One is extending TMA (or diethyl zinc (DEZ)) and water exposure time for each ALD cycle. Second is using a force flow chamber, a similar chamber structure with ref. [30], for ALD-MCP instead of a viscous flow reactor.

For conductive layer deposition, ZnO:Al_2_O_3_ ALD was performed using DEZ, TMA, and deionized water as Zn, Al, and oxidant precursor, respectively. Ultrahigh purity nitrogen was used as a carrier and purge gas. The Al_2_O_3_ ALD was performed using separate TMA and H_2_O exposures with sequence TMA/N_2_/H_2_O/N_2_ (4/35/4/35 s). And the ZnO ALD was performed using separate DEZ and H_2_O exposures, following the sequence: DEZ/N_2_/H_2_O/N_2_ (4/35/4/35 s), and the doping was performed by substituting TMA exposure for DEZ. The percentage of ZnO cycles was expressed by the quantity [DEZ/(DEZ + TMA)] × 100, where DEZ and TMA were the numbers of diethylzinc and trimethylaluminum pulses. The DEZ and H_2_O pulses alternated, and every fourth DEZ pulse was substituted with a TMA pulse. Consequently, 75% of the metal alkyl pulses were diethylzinc pulses. For a comparative study, pure Al_2_O_3_ as an emissive layer was used, and the coating thickness was approximately 8 nm for all MCPs. The thickness of ZnO:Al_2_O_3_ as a conductive layer was intentionally adjusted from 84 to 242 nm. The deposition temperatures of both conductive and SEE layers were 200°C. The detailed experimental parameters for conductive and SEE layers are listed in Table 1.

**Table 1:** **Detailed ALD experimental parameters for conductive and SEE layers**

**Description**		**Condition 1**	**Condition 2**	**Condition 3**	**Condition 4**
Conductive layer	Thickness (nm)	Approximately 84	Approximately 98	Approximately 115	Approximately 242
	ZnO/Al_2_O_3_ ratio	Percentage of ZnO cycles = 75%			
SEE layer		Pure Al_2_O_3_, approximately 8 nm			
Substrates		n-Si (100), glass, MCPs			
XRD analysis		Amorphous structure			
Other measurements		SEM, XPS, EDS, and electrical characterization for MCPs			

The surfaces of MCP samples were examined by scanning electron microscopy (SEM; Hitachi S4800, Hitachi, Ltd., Chiyoda, Tokyo, Japan). The film thickness and elemental composition were measured by cross-sectional SEM method and energy-dispersive X-ray spectroscopy (EDS; Oxford Aztec, Oxford Instruments, Oxfordshire, UK). The structure of thin films deposited on varied kinds of substrates was examined by X-ray diffraction (XRD; D8 ADVANCE from Bruker, Madison, USA). The surface chemical composition of samples prepared on silicon was measured by X-ray photoemission spectroscopy (XPS) at 4B9B beamline of Beijing Synchrotron Radiation Facility.

After ALD functionalization, the nickel chromium (NiCr) layer with 250 nm as electrodes was prepared on both MCP sides by electron beam evaporation system for MCP electrical characterization. The MCP resistances were measured using a Keithley Model 6517B electrometer under 10^−6^ Torr vacuum (Keithley, Cleveland, OH, USA).

**Figure 3:**
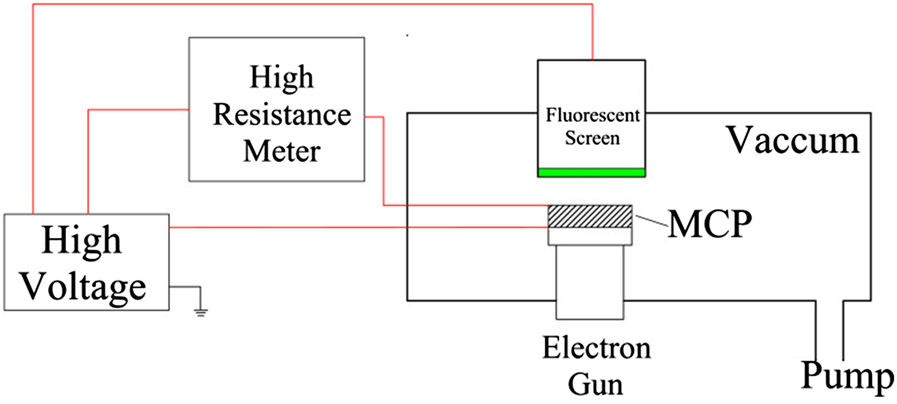
**Schematic diagram of MCP electrical measurement system.**

The schematic diagram of an MCP electrical measurement system is shown in Figure 3. A fluorescent screen containing green phosphor was lightening uniformly when ALD-MCP having good performance was bombarded by electron beam emitted from an electron gun.

## Results and discussion

**Figure 4:**
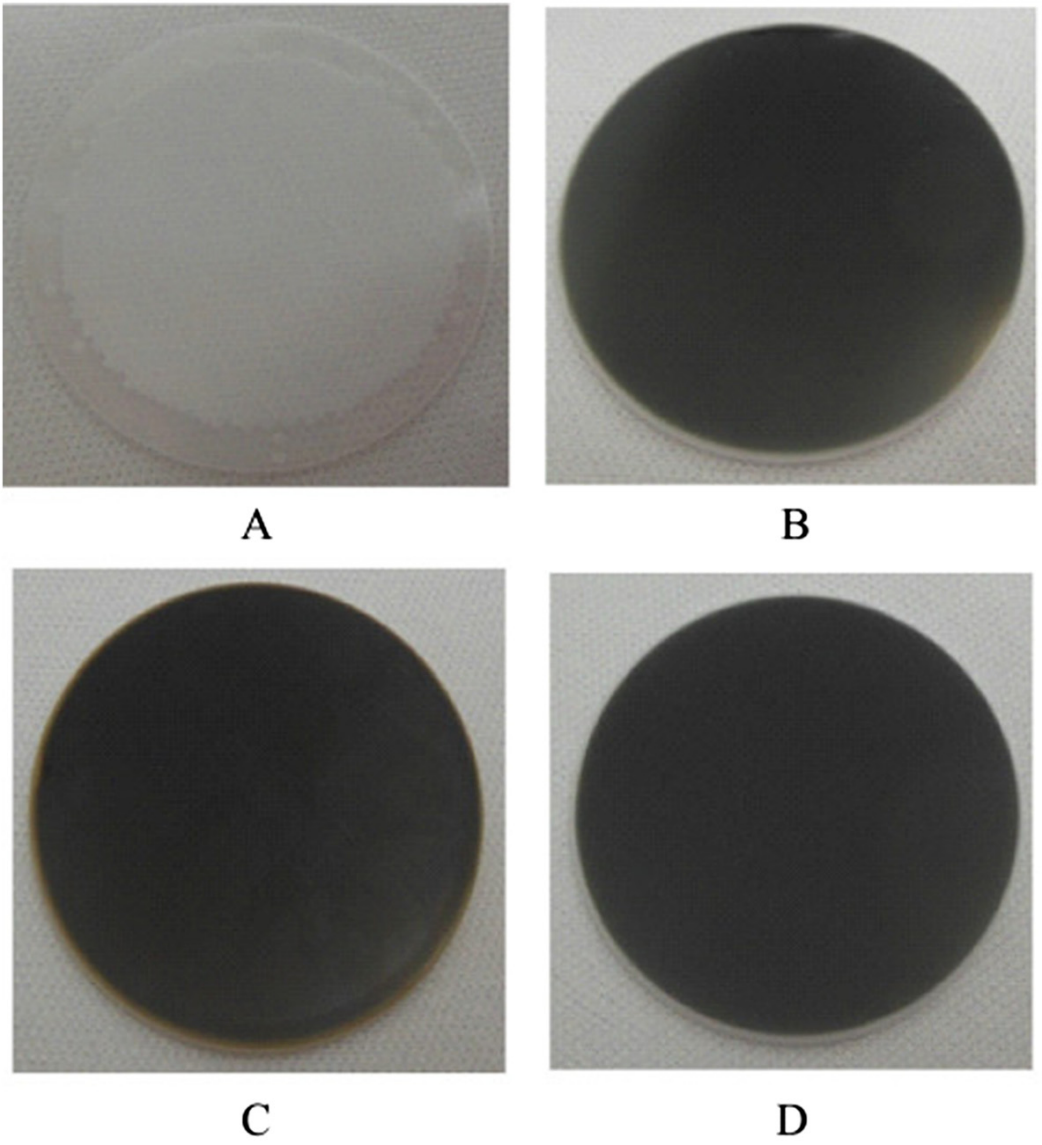
**Photographs of ALD-MCP samples at different process steps.** Pictures of bare 33 mm MCP **(A)**, ZnO:Al_2_O_3_ (84 nm)/Al_2_O_3_ (8 nm) deposition **(B)**, ZnO:Al_2_O_3_ (115 nm)/Al_2_O_3_ (8 nm) deposition **(C)**, and after 250-nm NiCr electrode deposition **(D)**.

Figure 4 shows the photographs of ALD-MCP samples at different process steps. For ALD functionalization, the bare MCP with 33 mm in diameter was used in this study as shown in Figure 4A. The main difference of samples made with conditions 1 to 4 is the film thickness of conductive layers, such as ZnO:Al_2_O_3_ (84 nm)/Al_2_O_3_ (8 nm) sample with condition 1 shown in Figure 4B and ZnO:Al_2_O_3_ (115 nm)/Al_2_O_3_ (8 nm) sample with condition 3 in Figure 4C. The other two samples show similar images like these (not shown here). Figure 4D shows the image of the sample with 250-nm NiCr electrode. The detailed analysis of these ALD-MCP samples including material characterization and device performance is given as follows.

### The studies of morphology, composition, chemical state, and structure of nano-oxide thin films prepared via atomic layer deposition

**Figure 5:**
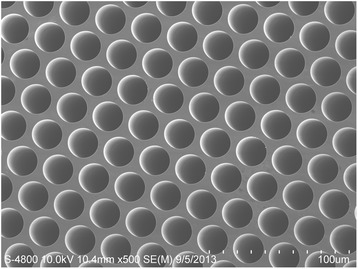
**Top-view SEM picture of MCP sample deposited with condition 1.**

Figure 5 shows the top-view SEM picture of nano-oxide thin films deposited on MCP with condition 1. And the other SEM images with conditions 2 to 4 look exactly the same which are not shown here. The uniform thickness of nano-oxide thin films and clear surface morphology of MCP are observed in the SEM pictures for all samples.

**Figure 6:**
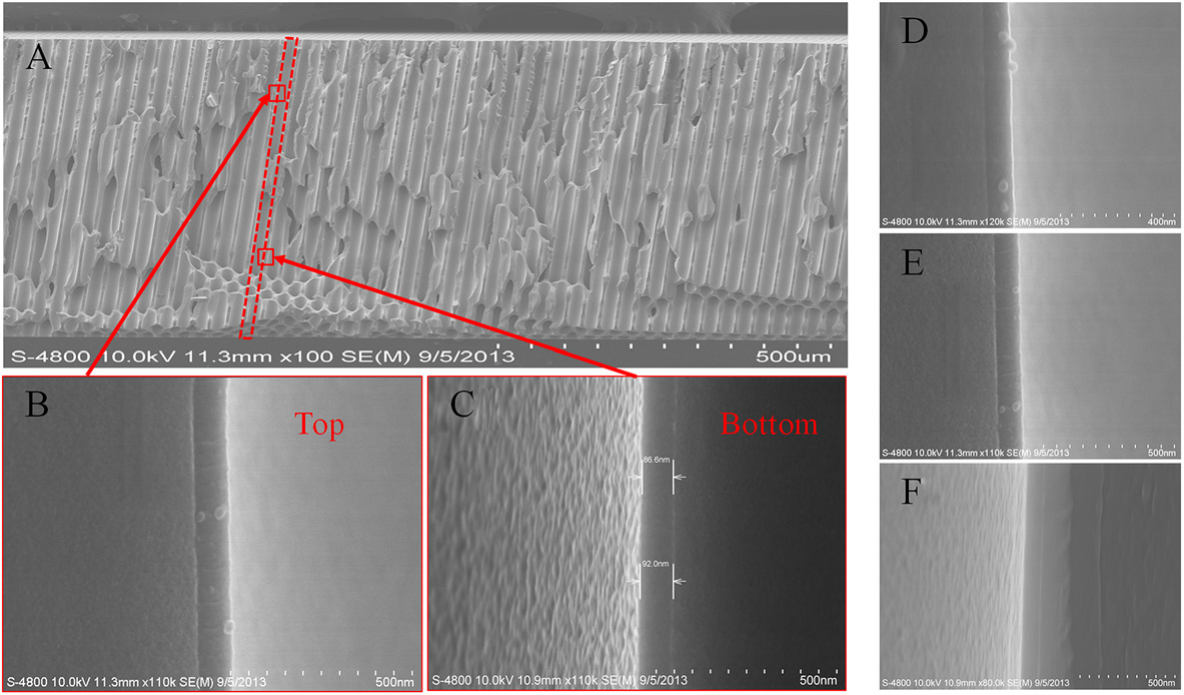
**Cross-sectional SEM pictures of ALD-MCP samples deposited on conditions 1, 2, 3, and 4.** SEM cross-sectional pictures of ALD-MCP samples deposited on condition 1 **(A)** with enlargement of the top part **(B)** and bottom part **(C)** in a pore and other samples on conditions 2 **(D)**, 3 **(E)**, and 4 **(F)**, respectively.

Usually, the most common approach to take thickness measurement is on the planar Si substrates as coupons instead on the MCP pores [7]. And it is difficult to use a SEM cross section to measure directly the thickness of nano-oxide thin films deposited on the inner surface of glass MCP pores because of difficulty of sample preparation. In this study, thickness was measured using a cross-sectional SEM method on MCP pores by careful sample preparation. Figure 6 shows the cross-sectional SEM pictures of ALD-MCP samples deposited on conditions 1 (A), 2 (D), 3 (E), and 4 (F), respectively. For showing thickness variation, clear cross sections at different locations in a pore were selected. For example, enlargements of the top part shown in Figure 6B and the bottom part shown in Figure 6C both in the same pore were chosen to take data points.

The total thicknesses of thin films including conductive and SEE layers deposited on the inner surface of MCP pores were measured accurately and summarized in Table 2. For each sample, eight thickness data including four in the top part and four in the bottom part of a pore were given carefully. And the average thicknesses of samples deposited on conditions 1 to 4 are 91.5, 105.8, 122.5, and 249.3 nm, respectively. In the meanwhile, the thickness uniformities, calculation formula shown in Table 2, for all samples are 2.26%, 1.53%, 1.34%, and 1.07% severally. The thickness uniformity of nano-oxide thin films deposited on the pore surface is worse than that of nano-oxide thin films deposited on the planar Si substrate. There are two reasons to explain this. One possible explanation is that the RMS surface roughness of the bare MCP pore is large due to complexity of glass MCP fabrication. Another is the difficulty of obtaining good cross-sectional SEM of MCP pores, such as a good one shown in Figure 6C and a poor one shown in Figure 6B.

**Table 2:** **Thickness variation of nano-oxide thin films in the MCP pores**

**Description**	**Thicknesses of coatings located in different locations of a pore (nm)**								**Average value (nm)**	**Uniformity (%)**
	**Top part**				**Bottom part**					
Condition 1	91.3	92.2	92.4	91.5	86.6	92.1	92.9	93	91.5	2.26
Condition 2	102.2	106.4	106.5	105.2	105.9	107.1	105.7	107.4	105.8	1.53
Condition 3	122.1	124.2	125.6	121.5	122.9	121.0	121.1	121.6	122.5	1.34
Condition 4	247.1	251.4	252.5	247.7	246.9	252.2	245.9	250.7	249.3	1.07

Uniformity = (Standard variation/Average variation) × 100%. Standard variation calculation formula: $S=\sqrt{\frac{1}{N-1}{\displaystyle {\sum}_{i=1}^N{\left({X}_i-\overline{X}\right)}^2}}$; average variation calculation formula: $\frac{X_1+{X}_2+\cdots {X}_N}{N}$.

According to the measured results, we can conclude that the nano-oxide thin films deposited on MCP pores by ALD technique with conditions 3 and 4 are more even than others. And the uniform thicknesses of conductive and SEE layers are crucial for MCP application.

**Figure 7:**
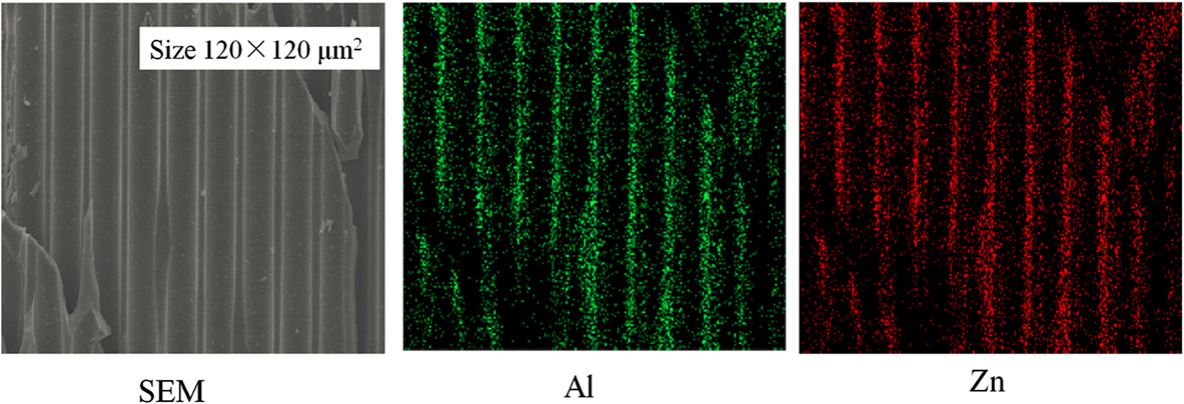
**EDS mapping of cross-sectional ALD-MCP prepared on condition 3.**

For analyzing the composition of nano-oxide thin films including conductive and SEE layers inside of the pores, EDS was used. The aluminum and zinc element distribution in the MCP pores with size 120 × 120 μm^2^ deposited with condition 3 is shown in Figure 7. From the figure presentation, the aluminum and zinc elements are uniformly distributed in the MCP pore surface. Similar results are obtained from other samples of EDS mapping characterization.

**Figure 8:**
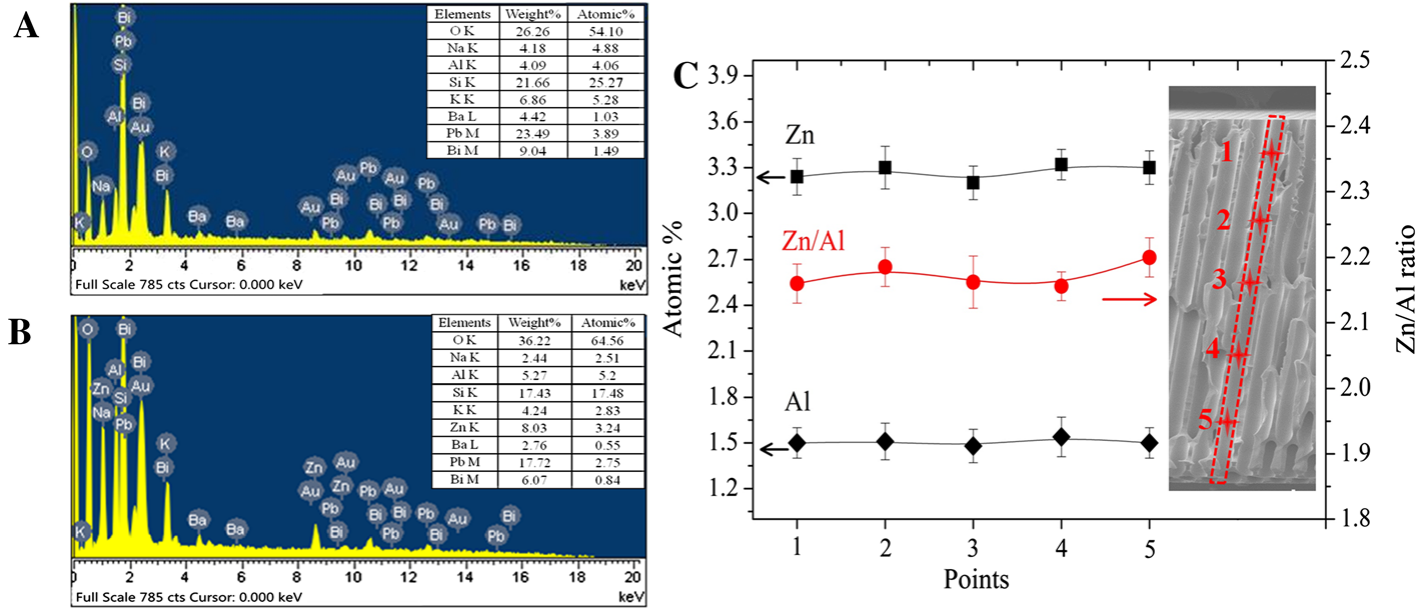
**Spectra and elemental composition of cross-sectional bare MCP and ALD-MCP samples.** Spectra and elemental composition of cross-sectional MCP samples: **(A)** bare MCP and **(B)** ALD-MCP with condition 3 and **(C)** its element distribution. The solid lines in (C) are guides to the eye.

Figure 8A,B shows the spectra and elemental composition of cross-sectional bare MCP and ALD-MCP samples, respectively. The detected Au element shown in both spectra is from the sputtering gold process in sample preparation. The bare MCP composition includes O, Na, Al, Si, K, Ba, Pb, and Bi elements not including Zn element compared to ALD-MCP sample. The Zn and Al atomic percentage are 3.24% and 5.2% in the ALD-MCP sample deposited on condition 3. The Zn element presence and the increase atomic percentage of Al element (from 4.06% in bare MCP to 5.2% in ALD-MCP) imply that the Al and Zn elements are introduced by an ALD growth process. The uniform composition of Al and Zn elements of ALD-MCP sample is characterized by EDS at five locations along the pore inner surface shown in Figure 8C. The atomic percentages of Zn and Al are relative values in bulk ALD-MCP materials, and the Al content excludes the influence of Al element in the bare MCP substrate. The results of Al and Zn contents at different locations signify that the elements are nearly uniformly distributed in the pore inner surface. This also implies that the ALD technique is capable of depositing homogeneous nano-oxide thin films on substrates with complex structure, and similar results were obtained in ref. [30-32].

The average value of Zn/Al atomic ratio is about 2.2 lower than the expected value of 3. There are two reasons for this. One is the existence of the ‘etching’ phenomenon which results in Zn element deficiency. Another more important is the influence of SEE layer using pure Al_2_O_3_ on the Al content that results in large Al atomic percentage and lower Zn/Al atomic ratio.

All of the ALD-deposited nano-oxide thin films on silicon substrates in this study have an amorphous structure according to the XRD analysis shown in Table 1. The amorphous structure of all samples probably resulted from low deposition temperature [33], high aluminum content [34], and thin thickness [35]. In Table 2, with nano-oxide thin film thicknesses increasing from 91.5 to 249.3 nm, the uniformities decrease slowly from 2.26% to 1.07%. This probably can be explained with the relationship between the structure and surface roughness of ZnO:Al_2_O_3_ film. The percentage of ZnO cycle is 75% in this study, and the XRD results show the amorphous structure for all samples suggesting that the ZnO nanocrystal growth is interrupted by the Al_2_O_3_ layers and the amorphous structure results in smoother surface when thickness increases [22].

**Figure 9:**
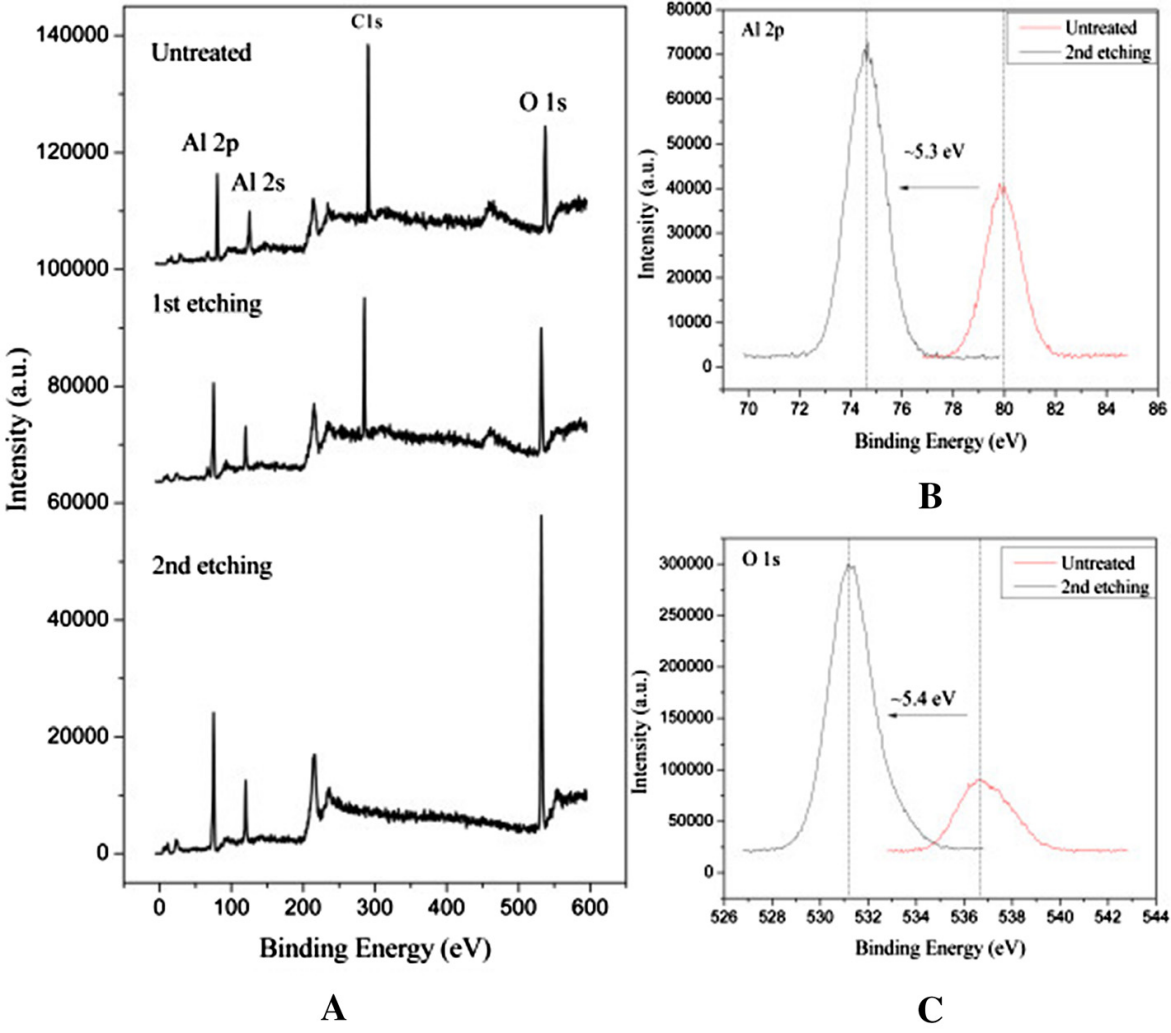
**XPS spectra of ALD-ZnO:Al**_**2**_ **O**_**3**_ **/Al**_**2**_ **O**_**3**_ **.** XPS spectra of ALD-ZnO:Al_2_O_3_/Al_2_O_3_ **(A)** as-deposited, first etching and second etching, and the peak shift before and second etching of **(B)** Al 2p and **(C)** O 1 s.

The XPS spectra of ZnO:Al_2_O_3_/Al_2_O_3_ sample deposited on silicon monitor coupon with condition 3 are shown in Figure 9. The peaks of C 1 s, Al 2p, Al 2 s, and O 1 s are appearing in 284.8, 79.9, 118, and 536.5 eV, respectively. The samples in this work contain some amount of carbon on the surface as shown in Figure 9A. For detecting the thickness of a carbon contamination layer, a 5-keV argon ion beam (Ar^+^) etching is performed for 10 min with a sample current of approximately 1.25 μA and raster scanned over a 1-cm^2^ area while lacking a neutralizing function. Under such conditions, a few nanometers thick layer is removed from the surface. After the first etching treatment using Ar^+^ bombardment, the peak of C 1 s still exists in the thickness of approximately 2.5 nm of the aluminum oxide. After the second etching treatment with thickness of approximately 2.5 nm, the C 1 s peak disappears and only the pure Al_2_O_3_ peaks are left. Therefore, the total thickness of thin films containing carbon atoms is approximately 5 nm. This is most likely due to the adsorbed atmospheric carbon [36] but may also result from remnants of the organic ALD precursor molecules that may diffuse to the surface under electron bombardment [37]. The containment of carbon on the aluminum oxide surface may have negative effects on the secondary electron emission and also the electrical performance.

The shift to lower binding energy of Al 2p and O 1 s peaks after etching treatment is 5.3 eV as shown in Figure 9B and 5.4 eV as shown in Figure 9C, respectively. The results imply that the charge effect is obviously existing when Ar^+^ is etching the surface without a neutralizing gun.

### Electrical performance of ALD-MCP with NiCr electrode

**Figure 10:**
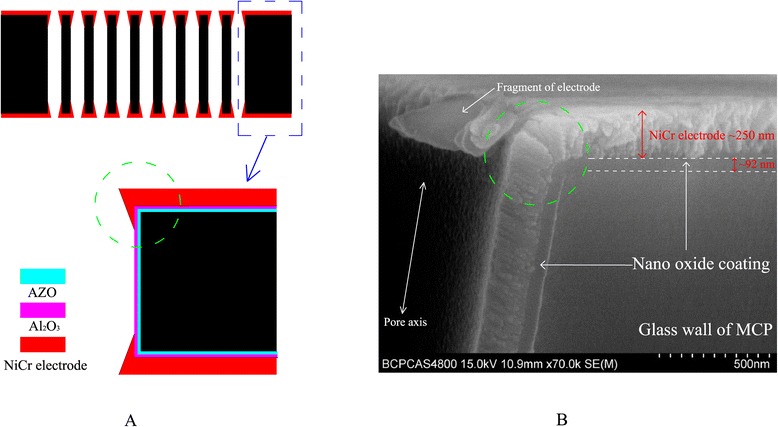
**Schematic diagram of cross-sectional ALD-MCP. (A)** Top: schematic diagram of cross-sectional ALD-MCP with electrode coatings; bottom: partial enlargement. **(B)** Cross-sectional SEM picture.

The NiCr electrode should be prepared on both sides of the MCPs prior to electrical characterization. Figure 10A top shows a schematic diagram of cross-sectional ALD-MCP with electrode coatings, and Figure 10A bottom shows a partial enlargement image. Please note that the bias angle of MCP depicted in Figure 10A is zero for simplicity. And a corresponding cross-sectional SEM picture is shown in Figure 10B. The end spoiling that the NiCr electrode penetrated into the pore is confirmed in Figure 10B, and uniform nano-oxide coating along the pore surface is visible. The thicknesses of NiCr electrode and nano-oxide coatings are approximately 250 nm and approximately 92 nm, respectively. The pore axis shows that the bias angle is 8° for the MCP used in this study.

**Figure 11:**
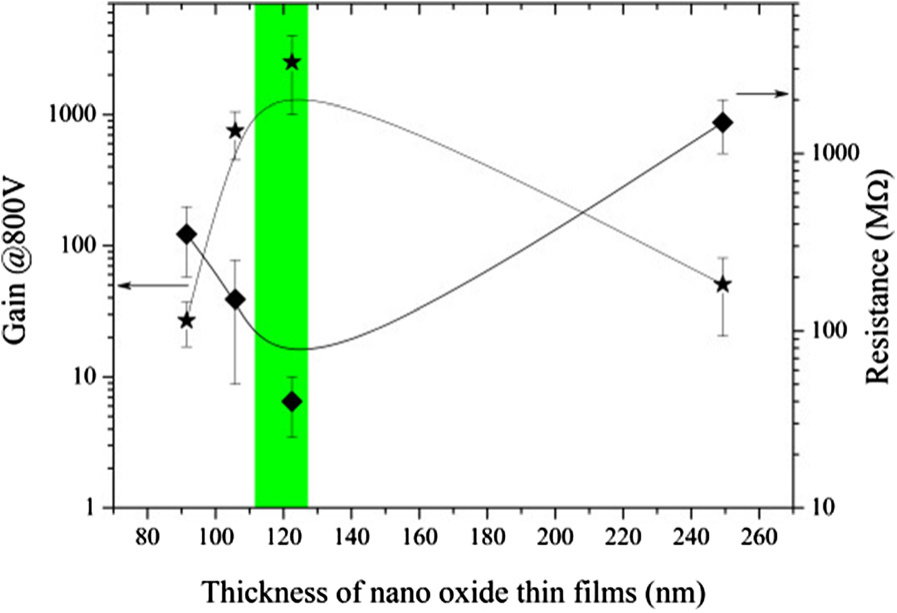
**Gain and resistance of ALD-MCP as a function of nano-oxide thin film thickness.** The solid lines are guides to the eye.

Figure 11 shows the gain and resistance of ALD-MCP as a function of nano-oxide thin film thickness. With the thickness increase, the gain increases quickly and reaches to a maximum value above 2,000 at DC 800 V with nano-oxide thin film thickness approximately 122 nm, then it decreases slowly with the thickness further increasing. According to fluorescent screen measurement, the screen can be uniformly lightening and displaying green light with the thickness range from 112 to 126 nm shown in the blue region in Figure 11.

**Figure 12:**
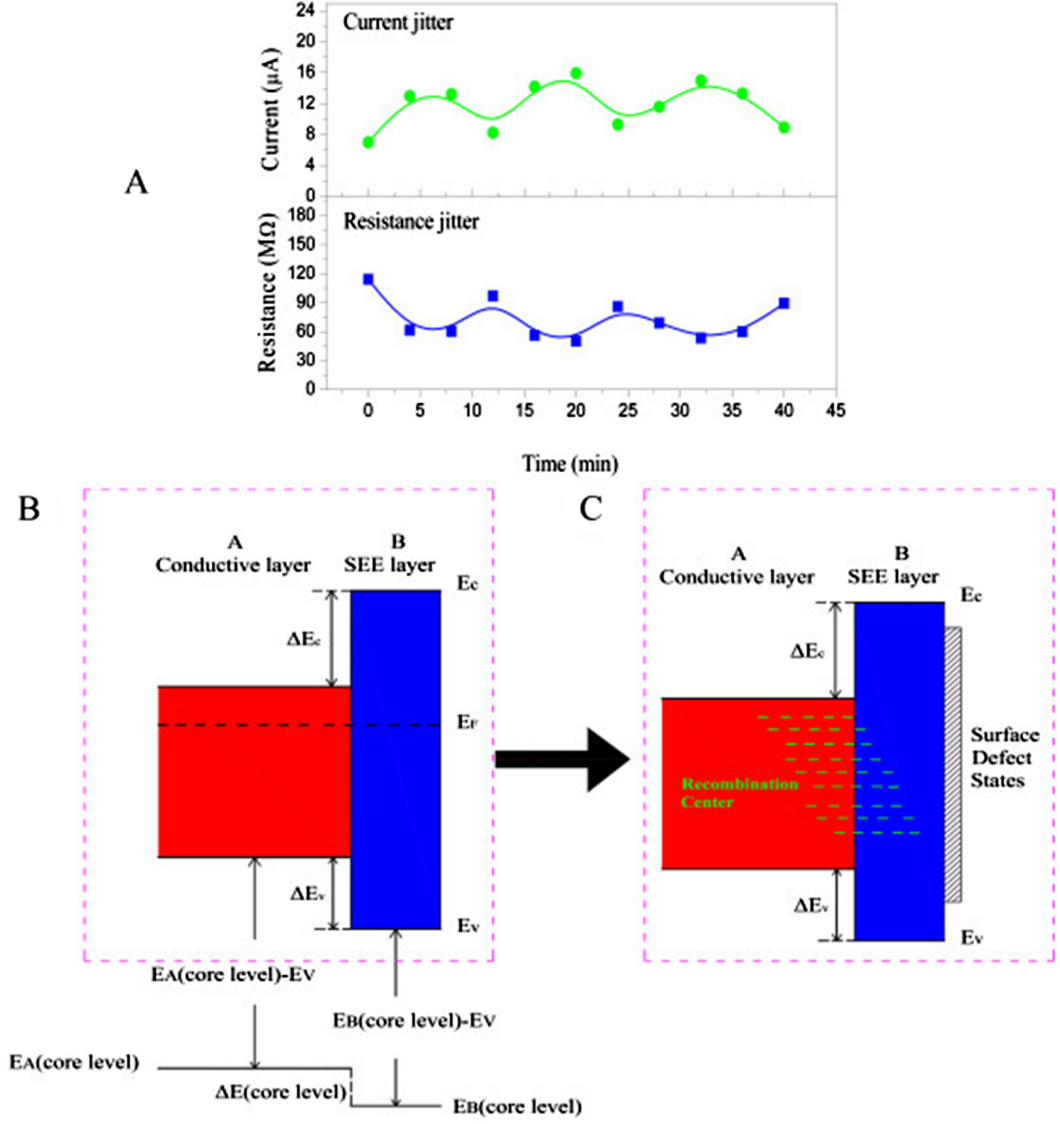
**Current jitter phenomenon and ideal and actual band gap diagram.** Current jitter phenomenon **(A)** and ideal **(B)** and actual **(C)** band gap diagram of conductive layer ZnO:Al_2_O_3_ and SEE layer Al_2_O_3_. The solid lines in (A) are guides to the eye.

From the gain and resistance results shown in Figure 11, the film prepared on condition 4 with the highest resistance also has one of the lowest gains. This could be caused by saturation of the MCP since the more conductive coatings will take longer to replenish charge. Indeed, the current (or gain) saturation is determined by the ability of pores to recharge and is mostly governed by the resistance of the conductive layer. Although electron scrubbing process varied substantially, the output currents for conventional MCP are exceeding 5% ~ 6% than strip currents when gain saturation appears, and for ALD-MCP, Beaulieu et al. observed that the gain saturation appeared at output currents equal to approximately 10% to 30% of strip currents [24]. And we consider that gain saturation of MCP with more conductive coatings appeared is a possible reason for the results of condition 4 shown in Figure 11. But the current jitter phenomenon shown below in Figure 12 is maybe not only related with gain saturation but also related with other reasons like Fermi level difference of materials and varied resistance coefficient of nano-oxide thin films that will be elucidated by further studies.

And we also observed current jitter phenomenon shown in Figure 12A during electrical measurement which is equivalent to resistance jitter. To our knowledge, this phenomenon has not been reported and the mechanism of this phenomenon is not fully understood. But we have considered possible reasons to explain this phenomenon.

Figure 12B shows an ideal band gap diagram of conductive and SEE layers. Without carbon atom contamination, the junction of conductive and SEE layer presents a typical band gap diagram. With carbon atom contamination, the recombination center and surface defect states appear, and the different band gap diagram is shown in Figure 12C. According to generalized Ohm’s law [38], the flow of charge is caused by Fermi level difference which is related to working temperature, and the valence band gap offset is influenced by temperature change and defect states produced by carbon atom contamination. Current jitter is probably associated with temperature change when MCP channels are bombarded by multiplied electrons. Moreover, resistance coefficient of nano-oxide thin films increases with temperature. So, the material resistance is changing with temperature. However, the relationship of Fermi level difference and resistance coefficient of nano-oxide thin films in MCP pores as a function of temperature variation is not present in this work and is studied on the way.

## Conclusions

The morphology, composition, chemical state, and structure of nano-oxide thin films ZnO:Al_2_O_3_ and Al_2_O_3_ prepared via atomic layer deposition were investigated. The nano-oxide thin film thickness uniformities for all MCP samples were less than 3%. The results of Al and Zn contents at different locations along the pore surface signified that the elements were nearly uniformly distributed. The results implied that the ALD technique was capable of depositing homogeneous nano-oxide thin films on substrates with a complex structure, such as glass MCP. The electrical properties of the MCP device as a function of nano-oxide thin film thickness were firstly studied. The electrical measurement results showed that the average gain of MCP was greater than 2,000 at DC 800 V with nano-oxide thin film thickness approximately 122 nm. And current jitter phenomenon was observed in this study. The steady resistance coefficient of nano-oxide thin films and free of contamination production process are probably important for eliminating current jitter. The mechanism of this phenomenon should be studied further.
